# Homicide and Suicide in the Elderly

**DOI:** 10.1192/j.eurpsy.2022.122

**Published:** 2022-09-01

**Authors:** P. Zeppegno, C.M. Gramaglia

**Affiliations:** 1 Amedeo Avogadro University of Eastern Piedmont, Medicina Traslazionale, Novara, Italy; 2 Università del Piemonte Orientale, Medicina Traslazionale, Novara, Italy

**Keywords:** Suicide, Homicide, Older Adults, risk factors

## Abstract

Homicide and suicide are complex phenomena raising questions and interest which go far beyond the medical and psychiatric field, as they represent a challenge for an understanding which is, first of all, human. In older adults, homicide and suicide may present together in the homicide-suicide phenomenon. The most common motive underlying this behavior in intimate partner relationships is the so-called “mercy killing”, where the perpetrator kills the partner to eventually allow relief from declining health conditions, and then commits suicide. Actually, older adults account for a disproportionately high number of suicide deaths and approximately 55% of late-life suicides are associated with physical illness, notwithstanding psychiatric comorbidity. Physical illness is more likely to eventually lead to suicidal behaviour when it represents a threaten for the individual’s independence, autonomy, self-esteem and dignity, and when it impacts on quality of and pleasure with life, sense of meaning, usefulness and purpose in life. As the current historical period is one marked by opportunities which have allowed a rapid increase of life expectancy and longevity, it clearly emerges the need to balance benefits and harms of curative and palliative therapies, especially for painful, terminal illnesses. The expression of suicidal thoughts in older adults, as well as behaviours suggesting “silent” or indirect suicidal attitudes, should be carefully investigated and clinicians should try to decode the possible communicative role of suicidal behaviour while avoiding premature conclusions about the “rationality” of patients’ decision to die.

**Disclosure:**

No significant relationships.

